# Association between sleep duration and burnout in healthcare professionals: a cross-sectional survey

**DOI:** 10.3389/fpubh.2023.1268164

**Published:** 2024-01-10

**Authors:** Jacksaint Saintila, Anderson N. Soriano-Moreno, Cristian Ramos-Vera, Susan M. Oblitas-Guerrero, Yaquelin E. Calizaya-Milla

**Affiliations:** ^1^Escuela de Medicina Humana, Universidad Señor de Sipán, Chiclayo, Peru; ^2^Clinical and Epidemiological Research Unit, School of Medicine, Universidad Peruana Unión, Lima, Peru; ^3^Área de Investigación, Universidad Cesar Vallejo (UCV), Lima, Peru; ^4^Research Group for Nutrition and Lifestyle, School of Nutrition, Universidad Peruana Unión, Lima, Peru

**Keywords:** sleep duration, burnout, healthcare professionals, occupational burnout, Peru

## Abstract

**Background:**

Short sleep duration in healthcare professionals is a recurring concern among researchers. On the other hand, the prevalence of burnout in this population group is experiencing exponential growth. Therefore, this study aimed to explore the association between sleep duration and burnout in healthcare professionals.

**Methods:**

This is a cross-sectional study. Data were collected by applying a non-probabilistic convenience sampling, considering a sample of 300 healthcare professionals from the public sector in Peru. The association between variables was explored using multivariate logistic regression. Values of *p* < 0.05 were considered statistically significant.

**Results:**

The results of the analysis in the crude models revealed that both men and women who slept < 7 h during workdays and days off were 8.33 (95% CI = 2.68–13.99, *p* = 0.004) and 17.18 (95% CI = 10.50–23.87, *p* < 0.001) times more likely to have burnout compared to those who reported ≥7 h, respectively. After adjusting for confounding variables, the association remained statistically significant.

**Conclusion:**

The findings of this study underscore the critical importance of sleep duration in the incidence of burnout among healthcare professionals. In the context of the global challenges to the mental and physical health of these professionals, our results highlight the urgent need to implement strategies at the organizational and individual level. This includes promoting a better work-life balance, and effective stress management and improved sleep quality.

## Introduction

According to the World Health Organization (WHO), burnout is defined as a response to chronic stress experienced in the work environment (1). In addition, it is characterized by three main features that include (1) the feeling of burnout or loss of energy, where the person experiences a lack of physical and mental resources to cope with work demands; (2) negative feelings toward work, such as frustration, disillusionment, and general dissatisfaction with the work environment; and (3) psychological distance toward work, which is manifested as a negative attitude and emotional distancing toward work tasks and responsibilities (1). The WHO recognizes burnout as a significant occupational health problem and includes it in the latest 11th revision of the International Classification of Diseases (ICD-11) (1). In addition, the understanding and recognition of occupational burnout has been strengthened, providing a more precise definition and placing it within the category of work-related problems, specifically in the professional setting. As a result, burnout has been widely recognized as a serious health problem (2).

On the other hand, burnout is a frequent experience among healthcare professionals; and in many cases, it is observed to a greater extent than in the general population (3). In fact, there is an exponential growth in the prevalence of burnout in healthcare professionals. For example, a recent systematic review found a burnout rate between 4.3 and 90.4% among health care workers who were on the frontline during the COVID-19 pandemic (2). Similarly, the results of another study conducted during the pandemic reported that 62 and 21% of the professionals had medium and high burnout, respectively (4). The current study was conducted during the pandemic. In addition, according to a systematic review by Dijxhoorn et al., burnout rates in palliative healthcare professionals were reported to range from 3 to 66% (3). It is therefore important for healthcare managers to support healthcare personnel through the implementation of measures such as reducing workload and improving the work environment.

The consequences of burnout symptoms can be perceived at both the individual and organizational level; this is because, on the one hand, burnout symptoms affect the wellbeing of the staff, impacting on physical health, leading to chronic fatigue and muscle pain (5). Moreover, studies carried out on healthcare professionals and the general population show that burnout is associated with an increased risk of developing mental disorders, such as depression and anxiety (6, 7). Similarly, professionals with burnout tend to experience a decrease in their work performance (8), because they have difficulty concentrating, making decisions, and performing their tasks efficiently (9), which can lead to poor quality patient care (10). There is also evidence that burnout can negatively affect the personal quality of life of healthcare professionals (11). In addition, due to lack of concentration and mental agility (9), stressed professionals may be more prone to make mistakes and suffer work-related accidents (10, 12). On the other hand, burnout can lead to absenteeism and job abandonment in healthcare organizations (13, 14), either due to illness or the need to take time off to recover, which can lead to high turnover, staff shortages, and additional workload for those who remain (3, 15).

Sleep plays a fundamental role in the regeneration and recovery of the human body after daily activities (16). During sleep, the body engages in a series of vital processes that contribute to restoring and strengthening physical and mental health (17). In fact, the U.S. National Sleep Foundation sets recommendations of 7–9 h of sleep for young adults and 7–8 h of sleep for older adults (18). However, sleep disorders are one of the most common health conditions, and sleep deprivation affects individuals of all ages, with healthcare professionals being particularly susceptible (19). A recent social network survey study that evaluated 963 healthcare workers during the COVID-19 pandemic reported that the average sleep duration was 6.1 h and nearly 96% of participants reported poor sleep (20). Another similar study conducted in physicians and nurses reported a prevalence of sleep disorders of 41.6% (21).

Short sleep duration may be one of the causal factors of burnout among healthcare professionals. Several studies conducted in both the general population and healthcare professionals have shown that the few hours of sleep can have an impact on the onset of burnout (22–24). In fact, in a study conducted on medical workers, it was found that those with a short sleep duration (< 7 h) had a higher risk of burnout (22). However, another similar study that analyzed physicians in training reported no association between sleep hours and burnout (23). There are still discrepancies or inconsistencies in the findings, which highlights the need for further research to gain a more complete understanding of the relationship between sleep duration and burnout in healthcare professionals.

On the other hand, investigating the effects of sleep duration on burnout is important in practical terms. However, studies examining the association between these variables in healthcare professionals are scarce, especially in the context of developing countries such as Peru. Understanding this association can provide valuable information to healthcare professionals and healthcare providers, allowing them to identify possible measures to reduce or prevent burnout, implement strategies compatible with a work environment favorable to adequate rest, mental health, and quality of life of healthcare professionals, which in turn can have a positive impact on the quality of healthcare provided. These results can be achieved through interventions and strategies that include work stress management programs and promotion of healthy sleep habits. Therefore, the aim of this study was to determine the association between hours of sleep and burnout in healthcare professionals in Peru.

## Materials and methods

### Study design and participants

This study has a descriptive cross-sectional design. The study included a sample of 300 healthcare professionals, both men and women, working in a public sector hospital located in the city of Rioja, in the Department of San Martin, Peru. The selection of participants was carried out by non-probability convenience sampling. In this case, the participants were not selected randomly, but according to their availability and convenience to participate in the study (25).

Data collection was carried out between October and December 2021 using a survey that included the following characteristics: (a) sociodemographic information including variables such as age, years of experience, level of weekly physical activity, among others, (b) anthropometric data, such as weight and height, and (c) a scale to assess burnout. In addition, the following inclusion criteria were established for the selection of participants: (a) to be Peruvian healthcare professionals and (b) to have at least 1 year of work experience. As for the exclusion criteria, the following were considered: (a) being in professional training, (b) lack of consent to participate in the study, and (c) incomplete completion of the survey.

### Ethical considerations

Before starting data collection, participants were provided with a detailed explanation of the study, including its purpose and procedures. It was emphasized that participation in the study was completely voluntary and that participants had the right to withdraw at any time without negative consequences. Once the relevant information was provided, written informed consent was obtained as a requirement for participation in the study. The research project was submitted for evaluation and was subsequently approved by the Research Ethics Committee of the Hospital II-1 de Rioja, obtaining approval the number CEP-080616. Finally, all procedures carried out in the development of this study were performed following the ethical principles established in the 1975 Declaration of Helsinki and its subsequent revisions.

### Variable measurements

#### Sociodemographic data

The measurement of sociodemographic data was performed using a registration form that included the following factors: gender, age, place of origin, profession, as well as anthropometric factors such as weight, height, and body mass index (BMI). BMI was classified according to WHO recommendations as follows: (a) < 18.5, underweight; (b) 18.5–24.9 kg/m^2^, normal; (c) 25.0–29.9 kg/m^2^, overweight; and (d) ≥30 kg/m^2^, obese (26).

#### Sleep duration

On the other hand, the duration of sleep was assessed by the following question: How many hours do you sleep during workdays and days off? The response alternatives were; (1) < 7 h = short sleep and (2) ≥7 h = adequate sleep. We did not use a questionnaire *per se*; this, due to the time constraints in which we had contact with health professionals during the day, therefore, only these essential and relevant questions were formulated to answer the research question. It is worth mentioning that previous studies have used similar questions to assess sleep duration (22, 27–29).

#### Burnout

In this study, a scale adapted and validated in the Peruvian population (30), based on the Maslach Burnout Inventory (MBI) (31), was used. After carrying out the validation of the instrument, a reliability value of 0.95 was obtained using Cronbach's alpha coefficient. The scale consists of 22 items and uses a 7-point Likert-type response scale, where 0 represents “never” and six represents “every day.” Additionally, the instrument consists of three dimensions: emotional exhaustion (nine items), depersonalization (five items), and self-fulfillment (eight items). The minimum score is 0 and the maximum is 132, the higher the score, the higher the level of work stress. The MBI was originally developed by Maslach et al. (31) in 1981 and has become a widely recognized and validated tool for assessing burnout in a variety of work contexts, including the healthcare setting.

### Statistical analysis

We describe categorical and numerical characteristics using frequencies and median and interquartile range, respectively. Fisher's exact test and the Wilcoxon rank sum test were used to explore differences in characteristics according to hours of sleep. Student's *t*-test was used to assess whether the level of stress was different according to the hours of sleep. Finally, we used linear regression to evaluate the association between hours of sleep and burnout level. We created a crude regression model and a multivariable regression model. In the multivariable model, we adjusted for potentially confounding variables according to epidemiological criteria. We considered a *p*-value of < 0.05 as significant. Analyses were performed with the R statistical program (www.r-project.org).

## Results

About 55% of the participants were men, with a median age of 34 years and an interquartile range of 29–40 years. Forty-five percent of the participants came from the jungle region and 34% from the coast. Participants reporting high burnout represented 21.7% of the sample. Approximately 39% of the participants reported sleeping < 7 h. More than half (58%) of the participants had normal nutritional status according to BMI, while excess body weight (overweight and obesity) accounted for 41%. The largest proportion of participants (33%) were nurses, followed by 31% who were physicians (Table 1).

**Table 1:** Characteristics of the sample studied.

**Characteristics**	***N* = 300^a^**
**Gender**	
Female	135 (45%)
Male	165 (55%)
Age	34 (29, 40)
**Place of origin**	
Coast	102 (34%)
Highlands	62 (21%)
Jungle	136 (45%)
**Sleep duration**	
< 7 h	116 (39%)
≥7 h	184 (61%)
**Burnout**	
Low	51 (16.6%)
Medium	186 (61.7%)
High	63 (21.7%)
BMI	24.52 (22.66, 25.95)
**Nutritional status**	
Underweight	2 (0.7%)
Normal	175 (58%)
Overweight	111 (37%)
Obesity	12 (4.0%)
**Profession**	
Physician	92 (31%)
Nurse	98 (33%)
Nutritionist	33 (11%)
Technical staff	77 (26%)

^a^n (%); Median (interquartile range).

Table 2 presents the characteristics of the participants according to sleep duration. Significant differences were found in the age distribution, with those who had a median age of 37 years with an interquartile range of 29–40 years sleeping ≥7 h, compared to those who were 32 years old with an interquartile range of 28–38 years (*p* < 0.001).

**Table 2:** Characteristics of the sample studied according to sleep duration.

**Characteristics**	**Sleep duration**		
	<**7 h**	≥**7 h**	***P*** **-value** ^b^
	***N*** = **184**^a^	***N*** = **112**^a^	
Gender			0.317
Male	48 (36%)	87 (64%)	
Female	68 (41%)	97 (59%)	
Age	32 (28, 38)	37 (30, 42)	< 0.001
Place of origin			0.797
Coast	39 (38%)	63 (62%)	
Highlands	22 (35%)	40 (65%)	
Jungle	55 (40%)	81 (60%)	
BMI	24.44 (22.68, 25.85)	24.61 (22.66, 25.99)	0.927
Nutritional status			0.612
Underweight	0 (0%)	2 (100%)	
Normal	70 (40%)	105 (60%)	
Overweight	3 (25%)	9 (75%)	
Obesity	43 (39%)	68 (61%)	
Profession			0.481
Physician	37 (40%)	55 (60%)	
Nurse	33 (34%)	65 (66%)	
Nutritionist	16 (48%)	17 (52%)	
Technical staff	30 (39%)	47 (61%)	

^a^n (%); Median (IQR).

^b^Fisher's exact test; Kruskal-Wallis rank sum test.

**Figure 1:**
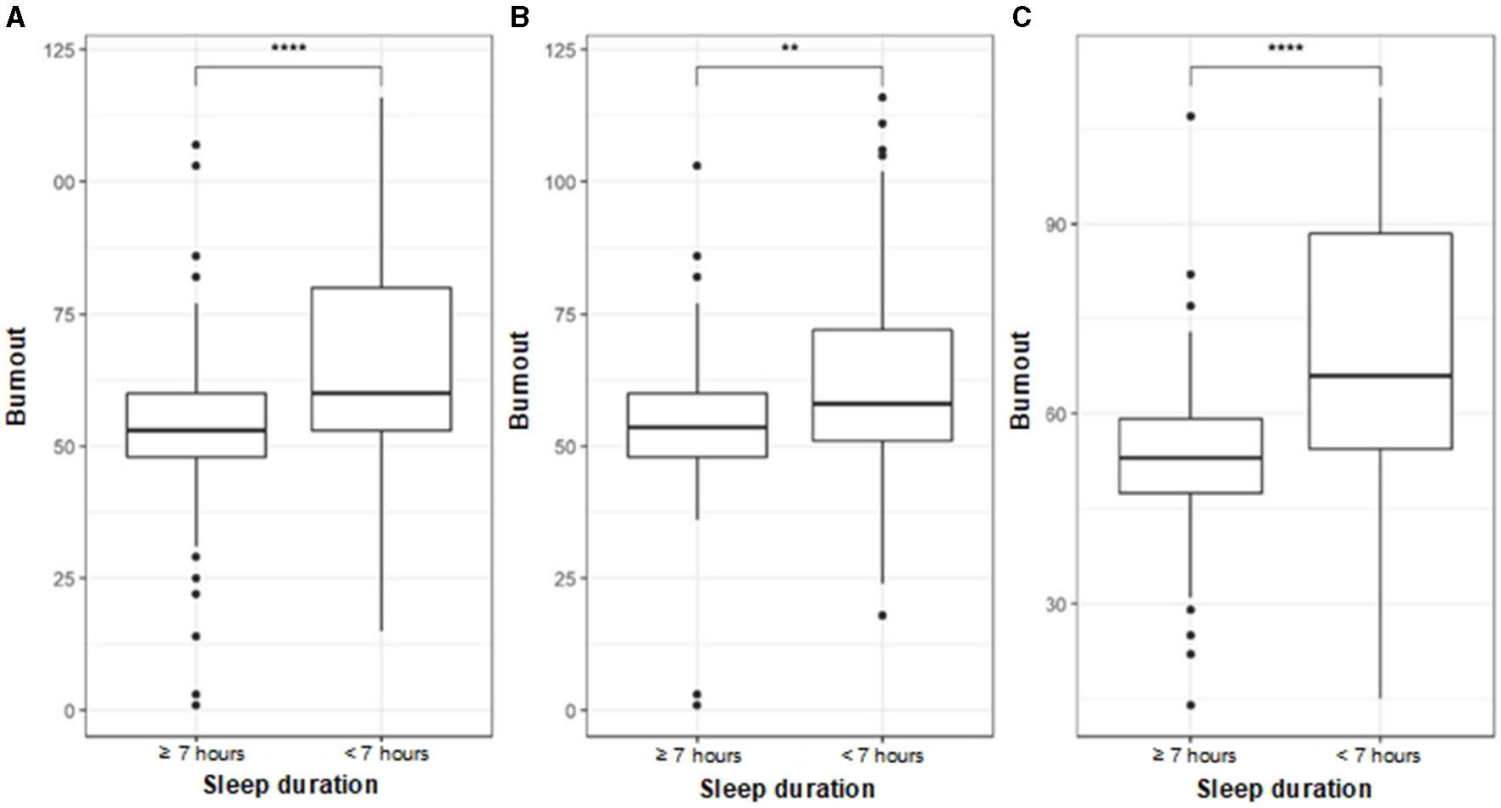
Comparison of stress level according to sleep duration. **(A)** All. **(B)** Male. **(C)** Female. Student's *t*-test was used to evaluate statistical differences. *p* < 0.01** and *p* < 0.0001****.

In Figure 1, we plot the level of burnout according to the duration of sleep and considering the gender of the participants. We observed that, for the participants as a whole, those participants who reported < 7 h had a higher level of burnout (*p* < 0.0001). When comparing between genders, it was observed that both men and women who slept < 7 h had a higher level of burnout. These results were statistically significant (*p* < 0.01 and *p* < 0.001, respectively).

Table 3 shows that in both the crude models and those adjusted for confounding variables, a significant association was found between the score obtained on the burnout scale. More specifically, in the crude models, it was observed that participants who reported < 7 h of sleep had 12.46 (95% CI = 8.11–16.80) more burnout points compared to those who slept ≥7 h. When comparing between genders, it was observed that men who slept < 7 h were 8.33 (95% CI = 2.68–13.99) times more likely to have burnout compared to those who reported ≥7 h. In relation to women, those who slept < 7 h were 17.18 (95% CI = 10.50–23.87) times more likely to have burnout compared to those who reported ≥7 h. After adjusting for confounding variables, the association remained statistically significant.

**Table 3:** Crude and adjusted linear regressions between sleep duration and burnout level.

	**Crude regression**			**Adjusted regression** ^**a**^		
All	B	95% CI	*p*	B	95% CI	*p*
≥7 h	1			1		
< 7 h	12.46	8.11–16.80	< 0.001	10.86	6.48–15.24	< 0.001
**Male**						
≥7 h	1			1		
< 7 h	8.33	2.68–13.99	0.004	6.62	0.57–12.67	0.032
**Female**						
≥7 h	1			1		
< 7 h	17.18	10.50–23.87	< 0.001	14.94	8.26–21.62	< 0.001

^a^The model that included all participants was adjusted for gender, age in years, origin, profession, and physical activity. The models for women and men were separately adjusted for the same variables, except for gender.

## Discussion

Burnout, also known as occupational burnout, is a common phenomenon in healthcare and can have a significant impact on the wellbeing of healthcare professionals (3). Among the various factors that may contribute to the development of burnout, sleep duration has emerged as an important aspect to consider (22, 24, 32). In Peru, a developing country with a poor health care system, as demonstrated by the COVID-19 pandemic (33), it is important to understand the association between sleep duration and burnout in healthcare professionals. The aim of the study was to determine the association between sleep duration and burnout among healthcare professionals. The relevant findings of the study were the following: (a) ~39% of participants reported a sleep duration of < 7 h, (b) those reporting high burnout represented 21.7% of the sample, (c) respondents with an age range of 29–40 years reported a sleep duration ≥7 h, compared to those with an interquartile range of 28–38 years, and (d) Both men and women who slept < 7 h were more likely to have burnout; particularly, the probability was higher in the case of women.

Current scientific evidence and recommendations of different national and international health and sleep organizations, such as the Peruvian Ministry of Health, the American Academy of Sleep Medicine, the Sleep Research Society, and the U.S. National Sleep Foundation agree that, in order to maintain optimal health, it is important to sleep between 7 and 9 h in a 24-h period (18, 34–36). Nevertheless, short sleep duration represents one of the most frequent health-related concerns affecting both the general population and healthcare professionals, although the latter are particularly susceptible (19). In the current study, 39% of participants reported < 7 h of sleep. Similar research in health and social care professionals found that 52.3% reported a particularly high prevalence of short sleep duration (37). In another study of physicians and nurses, a prevalence of sleep deprivation of 41.6% was reported (21). Similarly, the results of the current study are consistent with the findings of previous studies in healthcare workers, where an average sleep duration of 6.1 h was reported (20), less than recommended (18, 34–36). These findings could be due to the fact that professionals generally report insufficient sleep duration due to long working hours, rotating shifts, and the physical and emotional demands inherent to working in the health care field (37, 38). Inadequate sleep duration can decrease concentration, memory, and decision making, which can have important implications for patient safety and care due to medical errors (39–41). Therefore, it is necessary for both healthcare professionals and healthcare providers to recognize the importance of sleep and work together to address the challenges related to sleep duration and quality in the workplace.

On the other hand, burnout tends to be more common among health professionals than in the general population (3). In fact, the prevalence of burnout in healthcare professionals is experiencing exponential growth (2–4). In our study, 21.7% of the participants reported a high level of burnout. It is worth mentioning that the current study was conducted during the pandemic by COVID-19. Similarly, a wide variability was found in the burnout rate among health care workers during the COVID-19 pandemic, ranging from 4.3 to 90.4% (2). In addition, other studies reported that 62 and 21% of health professionals presented medium and high levels of burnout, respectively (4). Additionally, burnout rates in palliative care health professionals were reported to range from 3 to 66% (3). The COVID-19 pandemic has increased the level of burnout in healthcare professionals due to several factors, such as the performance of additional and complex work tasks related to an increased number of medical interventions, as well as the need to maintain stricter safety measures to prevent contagion (42). The consequences of burnout symptoms can be felt at both the individual and organizational level. On a personal level, they can lead to chronic fatigue and muscle pain (5), as well as mental disorders such as depression and anxiety (6, 7). In addition, professionals may experience a decrease in their work performance (8), difficulties in concentrating, making decisions, and performing their tasks efficiently (9), in addition to lack of concentration and mental agility (9), which makes them more prone to make mistakes and suffer occupational accidents (10, 12). At the organizational level, burnout can lead to absenteeism and job abandonment in healthcare organizations (13, 14), and result in poor quality patient care (10). Therefore, it is important to implement strategies at the individual and organizational level to reduce the risk of burnout and promote a healthy and productive work environment for healthcare professionals.

On the other hand, in this study we found that health professionals with an age range of 29–40 years reported a sleep duration ≥7 h, compared to those with an interquartile range of 28–38 years. These findings are consistent with the results of a study conducted in a group of physicians in a tertiary care hospital, where young physicians had an average sleep duration of 6.3 (6.0–6.7) h/night (43), which is < 7 h or more recommended by national and international organizations (18, 34–36). The situation of insufficient sleep among young physicians may be exacerbated by voluntary sleep restriction due to academic pressures and lifestyle factors, such as participating in nighttime recreational activities. The authors suggest that this may pose an additional risk to their health and wellbeing (43). Identifying the age groups most likely to experience insufficient sleep is essential to guide the design of interventions aimed at improving the duration and quality of sleep (44). With this, strategies can be implemented that specifically target younger healthcare professionals, as well as those in training, to address their unique challenges and promote healthier sleep habits.

Another relevant finding in the current study is that a statistically significant association was found between sleep duration and burnout. Sleep deprivation can have negative effects on mental health and emotional wellbeing, which may contribute to the development of burnout. This finding is supported by previous research in both healthcare professionals and the general population, where a significant association has been found between sleep duration and burnout (22, 24). For example, in a study of medical workers, it was found that those who slept < 7 h during workdays and days off had a higher risk of experiencing burnout (22). These findings are also echoed in another study that found that sleep duration was inversely related to job strain and burnout; as sleep duration decreased, job strain and burnout increased (32). It is important to mention that, in previous studies, it was shown that sleep loss and burnout caused medical errors and patient harm in ~37.7% of general practitioners and 39.9% of physicians in training (45). Therefore, encouraging adequate sleep duration may be critical to delaying and preventing burnout in the field.

It is worth mentioning that, in the crude logistic regression analysis and adjusted for confounding factors, in both men and women, it was observed that sleeping < 7 h represented a greater probability of presenting burnout. Moreover, in the particular case of women, we found that this probability was higher. In the context of the current study, this could explain, at least in part, the fact that the proportion of women reporting < 7 h of sleep was higher compared to men. In addition, differences in sleep between men and women are a well-documented phenomenon in the scientific literature (46, 47). In fact, for decades, various studies have reported that women tend to experience shorter sleep duration (48), more sleep-related symptoms (49), higher rates of insomnia (50), and lower rates of sleep apnea compared with men in the general population (51).

### Public health implications

Although these findings are cross-sectional in nature and do not allow us to establish causal relationships, the magnitude of the observed effects suggests that they could have public health relevance. The fact that those who sleep < 7 h are at greater risk of experiencing burnout may alert those responsible for human resources management in the hospital setting to the importance of promoting working conditions that allow workers to get adequate rest. In addition, the results highlight the relevance of addressing the wellbeing and mental health of healthcare professionals, given that prolonged burnout can negatively affect the quality of patient care and the safety of medical staff. Growing evidence in the scientific literature establishes an important connection between sleep quality and psychological wellbeing in healthcare professionals. Previous studies have shown that shorter sleep duration and lower sleep quality are significantly associated with an increased incidence of burnout among these professionals (22–24). Therefore, in the context of the global challenges to the mental and physical health of these professionals, this study highlights the need to implement preventive and supportive interventions to reduce the risk of burnout in this group of professionals to improve their quality of life and job performance. These measures could include workplace wellness programs, training in practical techniques for managing work stress, flexible work schedules, and promotion of a healthy work environment. It is also important to foster social support among healthcare professionals and provide resources for self-care and mental health promotion. By addressing these issues, it is hoped to mitigate the incidence of burnout and improve the overall wellbeing of healthcare professionals.

### Limitations

This study has several important limitations. First, the cross-sectional nature of the analysis precludes establishing causal relationships between sleep duration and burnout among health professionals. Given that the data were collected at a single point in time, it cannot determine whether sleep deprivation precedes the development of burnout or whether it is a consequence of burnout. Therefore, longitudinal studies that follow participants over time are required to gain a more complete understanding of this association. A second aspect to consider is the use of self-reported questionnaires to assess the variables under study, which could lead to response bias on the part of the participants (52). Particularly, in relation to the sleep duration variable, it is important to note that no specific questionnaire was used for its measurement. This was due to time constraints for interacting with the professionals during the day, so only one essential and relevant question was asked to address the research question. Therefore, the results related to sleep duration during workdays and days off should be interpreted with consideration of this methodological limitation. It is important to note that the use of a single question to assess sleep duration is not unusual in previous studies. In fact, several investigators have employed similar approaches to measure sleep duration in various populations (22, 27–29). Although these questions may not provide the same accuracy as a specific questionnaire, their usefulness lies in their simplicity and ease of administration, especially in situations where time and resources are limited (27, 28). A third limitation lies in the fact that, in the current study, sleep quality was not assessed nor was an objective method used to verify self-reported sleep duration. The evaluation of these aspects is especially important if we take into account that the perception of sleep quality may influence the self-assessment of sleep duration, which is a limitation to consider when interpreting the results of this research (22). Finally, we have used a relatively small sample; therefore, it may not be fully representative of all health professionals, which could limit the generalizability of the results to other populations. In this case, future research with larger and more diverse samples is recommended to better demonstrate the relationship between sleep duration and burnout in this group of professionals.

## Conclusion

The findings of this study revealed statistically significant association between sleep duration and burnout in both crude model regression analyses and those adjusted for potential confounders. The results persisted when comparing between genders, observing that both men and women who slept < 7 h had a higher probability of presenting burnout; particularly, the probability was higher in the case of women. The findings of this study underscore the critical importance of sleep duration in the incidence of burnout among healthcare professionals. In the context of the global challenges to the mental and physical health of these professionals, our results highlight the urgent need to implement strategies at the organizational and individual level. This includes policies aimed at promoting a better work-life balance, ensuring sufficient opportunities for rest and recuperation. In addition, initiatives that train healthcare professionals to effectively manage stress and improve sleep quality should be encouraged. Addressing these aspects is especially important to meet today's challenges in healthcare settings, ensuring the quality of patient care and the wellbeing of those on the front lines of healthcare.

## Data availability statement

The original contributions presented in the study are included in the article/supplementary material, further inquiries can be directed to the corresponding authors.

## Ethics statement

The studies involving humans were approved by Research Ethics Committee of the Hospital II-1 de Rioja. The studies were conducted in accordance with the local legislation and institutional requirements. The participants provided their written informed consent to participate in this study.

## Author contributions

JS: Conceptualization, Data curation, Formal analysis, Funding acquisition, Investigation, Methodology, Writing – original draft, Writing – review & editing. AS-M: Conceptualization, Data curation, Formal analysis, Funding acquisition, Investigation, Methodology, Writing – original draft, Writing – review & editing. YC-M: Conceptualization, Formal analysis, Methodology, Supervision, Writing – review & editing. CR-V: Investigation, Supervision, Validation, Visualization, Writing – review & editing. SO-G: Writing – review & editing.
